# Immune Control of PRRS: Lessons to be Learned and Possible Ways Forward

**DOI:** 10.3389/fvets.2014.00002

**Published:** 2014-10-14

**Authors:** Massimo Amadori, Elisabetta Razzuoli

**Affiliations:** ^1^Laboratory of Cellular Immunology, Istituto Zooprofilattico Sperimentale della Lombardia e dell’Emilia Romagna, Brescia, Italy

**Keywords:** pig, PRRSV, vaccination, immunomodulation, interferon-alpha

## Abstract

Porcine reproductive and respiratory syndrome (PRRS) is an elusive model of host/virus relationship in which disease is determined by virus pathogenicity, pig breed susceptibility and phenotype, microbial infectious pressure, and environmental conditions. The disease can be controlled by farm management programs, which can be supported by vaccination or conditioning of animals to circulating PRRS virus (PRRSV) strains. Yet, PRRS still represents a cause of heavy losses for the pig industry worldwide. Immunological control strategies are often compounded by poor and late development of adaptive immunity in both vaccinated and infected animals. Also, there is evidence that results of field trials can be worse than those of experimental studies in isolation facilities. Neutralizing antibody (NA) was shown to prevent PRRSV infection. Instead, the role of NA and adaptive immunity on the whole in virus clearance after established PRRSV infections is still contentious. Pigs eventually eliminate PRRSV infection, which may be correlated with an “educated,” innate immune response, which may also develop following vaccination. In addition to vaccination, an immunomodulation strategy for PRRS can be reasonably advocated in pig “problem” farms, where a substantial control of disease prevalence and disease-related losses is badly needed. This is not at odds with vaccination, which should be preferably restricted to PRRSV-free animals bound for PRRSV-infected farm units. Oral, low-dose, interferon-α treatments proved effective on farm for the control of respiratory and reproductive disease outbreaks, whereas the results were less clear in isolation facilities. Having in mind the crucial interaction between PRRSV and bacterial lipopolysaccharides for occurrence of respiratory disease, the strong control actions of low-dose type I interferons on the inflammatory response observed *in vitro* and *in vivo* probably underlie the rapid clinical responses observed in field trials.

## Introduction

There are two basic principles underlying the possible success of a vaccination strategy against viral diseases. On the one hand, a reasonable cause–effect relationship must be recognized between a virus agent and an infectious disease or an infectious syndrome, fulfilling whenever possible Koch’s postulates. On the other hand, having defined an etiological agent, a protective immune response must be generated after vaccination against structural and/or non-structural proteins (NSPs) of the virus in a naive host to prevent infection and/or disease occurrence. On the basis of the observed parameters (extent of infection and/or disease symptoms), protection may be defined as virological, clinical, or both. In this conceptual framework, effective vaccines have been developed for a plethora of viral agents in the veterinary field, leading, e.g., to the eradication of rinderpest (1). Along with such successful vaccines, major failures were experienced as well; among these, the example of African swine fever (2) is probably outstanding in terms of both disease importance and extent of the applied research efforts. Porcine reproductive and respiratory syndrome (PRRS) is probably in-between such extremes in vaccine history, whereby contradictory reports accumulated about PRRS vaccine efficacy, and various options are being evaluated to obtain more effective immunizing products. In hindsight, the two aforementioned requirements for a vaccine were neither consistently confirmed nor rejected for PRRS virus (PRRSV) by the scientific community, despite long and exhaustive research efforts worldwide (3). Therefore, after the first isolation of PRRSV in Europe in 1991, showing a formal respect of Koch’s postulates (4), consistent susceptibility to experimental infection was only shown in late pregnant sows. Therefore, many other attempts to reproduce disease symptoms were unsuccessful, and many uncertainties still exist about fundamental issues of the host/virus relationship in the PRRS model (5).

## The Disease

Porcine reproductive and respiratory syndrome emerged in the late 80s, in USA, and later on, in Europe, spread quickly and became enzootic in the pig population in most countries all over the world. Late-term reproductive failure in sows with transplacental transmission of the virus, preweaning mortality of piglets, respiratory distress, anorexia, and possible cutaneous hyperemia in weaners and growers are the most evident clinical signs of PRRS (6). PRRSV is currently one of the most important swine pathogens, causing heavy economic losses in pig farms all over the world. These were reckoned in the height of $ 560 million/year in USA (7), and a similar impact can be also envisaged in other countries with intensive pig farming activities. The impact is related to both disease occurrence (direct losses) and an increased prevalence of secondary infections and/or growth check. The causative agent is an enveloped, positive-strand RNA virus of the *Arteriviridae* family (8). Whereas PRRSV infection is present in the large majority of pig farms, the prevalence of both PRRS and PRRS-associated diseases is highly variable. Two swine arterivirus types have been identified, to date, as etiological agents: the European (EU) type I, with the first strain isolated in 1991 and named “Lelystad”; the North American type II, isolated in 1992 with the acronym ATCC VR-2332 (8). A recent map of the global distribution of type I and type II PRRSV can be found online at http://www.pig333.com/what_the_experts_say/introduction-dissemination-and-perpetuation-of-prrs-virus-in-a-region_6616/. There is only 50–60% sequence identity between the EU and North American types (9), which implies the existence of two distinct genotypes derived from a common ancestor (10). On the whole, virus infection and disease may differ between type I and type II PRRSV, the latter being more frequently associated with disease symptoms. As a result, infection and protection models were preferentially established for type II, as opposed to type I PRRSV. In particular, type I PRRSV is primarily a reproductive pathogen, whereas its direct role in respiratory disease under field conditions is ill defined (11). On the contrary, the virulence of the two PRRSV genotypes is not significantly different toward the male reproductive system (12). Disease control has been traditionally founded on a combination of management and biosecurity measures, generally aimed at “stabilization” of the herd as a priority, i.e. a condition in which clinical signs of PRRS are absent in the breeding-herd population, and PRRSV is no more transmitted from sows to their offspring (13). After reaching this preliminary status, eradication may be possible by herd closure (14), which is eased by proper air filtration devices to prevent airborne infection (15). In addition to management and biosecurity measures, various immunological approaches (vaccination and/or “conditioning” to circulating PRRSV strains) are also used for control of both respiratory and reproductive disease (6).

### Risk factors for disease occurrence

Although Koch’s postulates were formally fulfilled for PRRSV at the very beginning of PRRS history, most experimental infection studies failed to provoke overt disease (16), and virus is often found in clinically healthy pigs. Also, the findings of field studies defined PRRS as a multifactorial disease, in which PRRSV strains showed different features of pathogenicity and agonist interaction with both microbial and non-microbial environmental parameters, which are hardly reproducible under experimental conditions in isolation facilities (11).

The concurrence of microbial and non-microbial components for disease occurrence should be examined in detail. First, there is wide circumstantial evidence reported by swine practitioners that prevalence of respiratory disease is often related to hygiene and welfare conditions; the adjustment of basic housing conditions such as the animals’ concentration in the weaning crates can deeply affect respiratory disease morbidity and related losses. Second, disease occurrence is probably eased in intensive pig farms by the present lean type pig phenotypes and the synergism between PRRSV and bacterial Lipopolysaccharides (LPS); this underlies the occurrence of respiratory disease, as opposed to either PRRSV or LPS alone (17). The mechanism can be probably traced to PRRSV-driven activation of inflammosomes in LPS-primed macrophages through the small envelope protein E, which gives rise to increased IL-1β release (18). The consequences may be conceivably worse in lean type pigs, characterized by constitutive high levels of oxidative stress (19). This can definitely exacerbate the crucial synergism between PRRSV and LPS, because of the well-known process of inflammatory auto-amplification through nuclear factor-kappaB (NF-kB) activation by reactive oxygen metabolites (20). Thus, the heavy exposure of lean type pigs to air-driven LPS (21) in intensive farms further increases this kind of risk. Interestingly, there is evidence that PRRSV had circulated in Eastern Europe for a long time before the recognition of clinically overt PRRS (22), which seems to be temporarily connected with the advent of lean pig phenotypes in Western Europe in the 80s and their contact with the virus after the reunification of Germany in 1990. Non-lean pigs show a reduced susceptibility to PRRSV (23), as also shown by the comparative evaluation of PRRSV infection of a local German breed and of commercial Pietrain pigs (24). This latter feature is relevant to strong rising evidence of a genetic component in susceptibility to PRRSV (25). *In vitro*, the early induction of a type I interferon (IFN) response may underlie the reduced susceptibility of Landrace pig macrophages to PRRSV replication (26).

Finally, the age of pigs plays an important role, non-adult animals showing the greatest susceptibility to both infection and disease (27). Pregnant sows are consistently susceptible to reproductive infection in late pregnancy (72–93 days of gestation under experimental conditions), following the accumulation of highly susceptible macrophages in the placenta (28).

**Figure 1:**
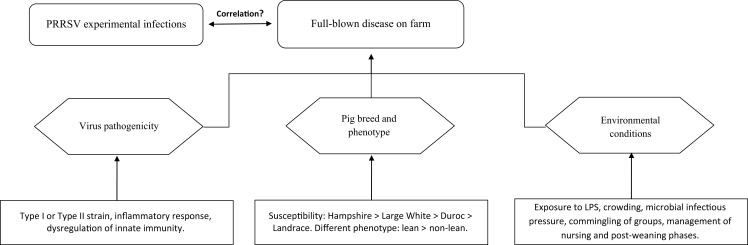
**Occurrence of clinically overt PRRS on farm**.

Owing to the above, disease occurrence in pigs of the same age is likely to be the product of three distinct components (Figure 1):

1. Virus pathogenicity (ill-defined to date).
2. Pig breed (susceptibility: Hampshire > Large White > Duroc > Landrace) and phenotype (lean > non-lean) (25).
3. Environmental conditions.

Any of the three above components may dramatically affect disease occurrence and/or prevalence on farm. Thus, PRRS may be ranging from subclinical to fatal disease with wide fluctuations of both morbidity and mortality, as well as of direct and indirect economic losses (6). The disease may be occasionally characterized by extreme virulence, as in the outbreaks sustained by the Chinese high fever virus, abortion “storm” viruses and some Belarus PRRSV strains (29–31). However, even for these strains, the synergism between PRRSV and bacterial LPS is likely to play a crucial role in amplifying the inflammatory response of infected macrophages (32).

## Biological Properties of PRRS Virus

Porcine reproductive and respiratory syndrome virus shows extreme variability of its nucleotide sequence, which may imply amino acid changes of major importance for diagnostic purposes (33).

There is strong evidence that PRRSV suppresses T-cell recognition of infected macrophages (34), which can be eased by the poor accessory features of porcine alveolar macrophages (PAM) (35). Distinct signs of immunosuppression are frequently observed in PRRSV-infected pigs, which can be conducive to an increased incidence and severity of secondary bacterial infections (36), and contribute to the “porcine respiratory disease complex” (PRDC).

As for the majority of PRSSV strains, there is evidence of an inadequate response of the innate immune system in the early phase of PRRSV infection, compared with other virus infections of swine, as well as of a late, erratic onset of neutralizing antibody (NA) and virus-specific IFN-γ responses (37). In the authors’ experience, the aforementioned delay observed under experimental conditions may well correspond in non-adult pigs to a substantial lack of a virus-specific IFN-γ response for a long time under field conditions (38), in agreement with the results obtained in non-adult pigs exposed to a type II PRRSV strain (27). An early, non-specific IFN-γ response during PRRSV infection may be observed as well (39, 40).

The main immunosuppressive features of PRRSV have been actively investigated to identify the structural and non-structural virus components exerting such activities. As for the downregulation of the type I IFN response, a main role was evidenced of PRRSV NSPs 1α, 1β, 2, 4, and 11, with effector mechanisms related to inactivation and block of nuclear translocation of interferon response factor (IRF) 3, interferon-stimulated gene factor 3 (ISGF3), and signal transducer and activator of transcription (STAT) 1, as well as to processing of ISG15 (an ubiquitin-like protein coded by the ISG15 gene) and IκB kinase (IKK)α (IFN response and NF-kB signaling, respectively) (41). In practice, there is evidence that multiple suppressive functions are exerted by NSPs, whereas further suppressive activities could be related to IL-10 production through the nucleocapsid (N) protein (41). These suppressive features may be counteracted by compensatory mechanisms related to the redundancy of the regulatory pathways in the immune system. In particular, as highlighted in a previous review (42), the final levels of IL-10 and IFN-β will be dependent on both PRRSV infection and LPS-driven activation of toll-like receptor 4 (TLR4); also, the final NF-kB signaling levels will be determined by N protein’s positive and NSP’s negative regulatory effects.

Immunosuppression can be also accounted for by T regulatory (Treg) cells, which develop following infection by type II PRRSV strains (43).

On the whole, the immunosuppressive impact of any PRRSV strain can be conveniently evaluated on the basis of the IFN and IL-10 responses both *in vivo* and *in vitro*. In this respect, the profiles of cytokine responses during PRRSV infection and the existence of outright “immunotypes” of PRRSV underlying such profiles (44) have been implied as possible pathogenicity factors. In particular, the property of PRRSV to induce and amplify an IL-10 response in infected pigs and the possible synergism with other microbial agents have been highlighted both *in vitro* and *in vivo* (45). Decreasing serum concentrations of IFN-γ and persisting IL-10 responses might cause upregulation of CD163 in macrophages (46), which might contribute to enhanced PRRSV replication and long duration of viremia.

The above features of most type I PRRSV strains differ from those of virulent EU subtype 3 and Chinese type II virulent strains, which induce instead IFN-α and inflammatory cytokine responses early after infection and persisting IL-10 responses later on, often correlated with a serious clinical outcome of PRRSV infection (47, 48). These features (IFN-α and IL-10 responses) were in agreement with the properties of a moderately virulent type I PRRSV strain, whereas an early IFN-γ response with cessation of viremia was shown after infection with an attenuated PRRSV strain under the same experimental conditions (40). Also, an early IFN-α response was shown after PRRSV infection of gilts at gestation day 85 (49). Such a response can be reproduced *in vitro* by some PRRSV strains in cultures of pig plasmacytoid dendritic cells (50). Most important, concomitant IL-10 and IFN responses can cause a gain of pro-inflammatory activity, as shown in human models of endotoxemia (51). Therefore, it is tempting to speculate that both extent and timing of the IL-10 response in PRRSV-infected animals are crucial in terms of inflammatory response and relevant clinical repercussions.

**Figure 2:**
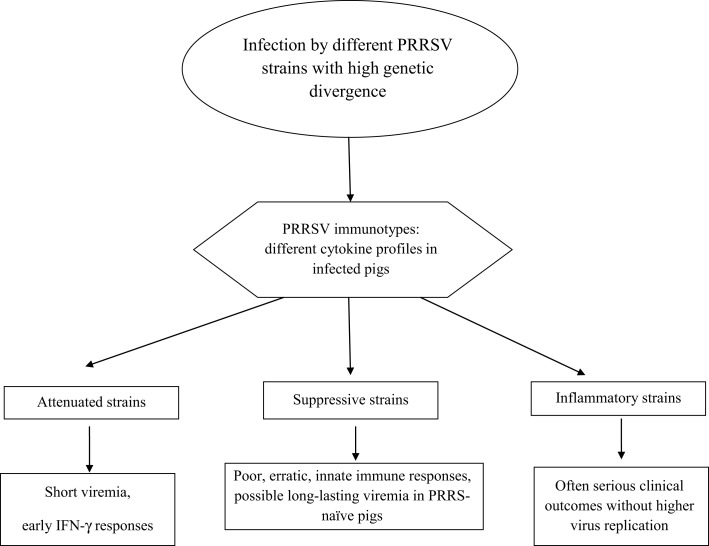
**PRRSV phenotypes**.

On the whole, PRRSV strains give rise to different cytokine responses fluctuating between the aforementioned, extreme virus phenotypes (Figure 2):

- **Attenuated**: short if any viremia is observed with an early, probably non-virus-specific IFN-γ response.
- **Suppressive**: these strains induce a delayed and erratic development of innate and adaptive immunity, often correlated with long-lasting viremia after infection of non-adult, PRRS-naïve pigs.
- **Inflammatory**: early, strong inflammatory cytokine responses with persisting IL-10 plasma levels are observed, which is often correlated with serious clinical signs. This virus phenotype may fulfill Koch’s postulates after both reproductive and respiratory infection of PRRS-naïve pigs.

In turn, the three virus phenotypes are in conceptual agreement with the aforementioned virus “immunotypes” shown *in vitro* (44).

## Mechanisms of Immune Protection and Virus Clearance

Whereas a cause/effect relationship between the swine arterivirus and PRRS can be proved for many type II strains, this is not the case for the vast majority of type I (EU) strains, which usually give rise to subclinical infections and relatively low levels of viremia under experimental conditions (3). Also, major discrepancies are reported for both EU and North American strains between experimental trials under controlled conditions and observations made in the field on farm (52). This highlights the complex pathogenesis of the disease, which underlies the accumulation of conflicting reports and sets of data related to both field trials and protocols of experimental infection.

Most important, the definition of clear correlates of immune protection is still controversial. The antibody response observed by ELISA is not protective and a fraction of these immunoglobulins have been associated with an immunopotentiating effect of PRRSV infection (53). The inhibition of antiviral cytokine responses through ligation of porcine FC gamma receptor I in pulmonary macrophages may account for the observed adverse effects (54), which could be also correlated with anti-idiotypic antibody responses (55). A few reports are available about the mucosal antibody response to PRRSV, which has been somewhat neglected by the scientific community. In the authors’ experience, the IgA antibody response to PRRSV in oral fluids during primary infection is concomitant with the serum antibody response, even if the duration tends to be shorter and followed by subsequent “waves” of antibody production (Amadori, Razzuoli, unpublished data).

Cytotoxic T lymphocytes do not eliminate PRRSV-infected macrophages (56) and antibody-dependent lysis by complement and phagocytic cells does not work since viral glycoproteins are not expressed on the plasma membrane (57). Accordingly, in a primary infection of PRRS-naive pigs, the decay of viremia is not dependent upon the adaptive immune response to PRRSV (37). In particular, the height and duration of viremia is not affected by the PRRSV-specific IFN-γ response in the first 3 weeks after challenge (58). The decay of viremia may be correlated with an early, non-virus-specific production of IFN-γ by activated natural killer (NK) cells (39). Instead, in a later phase (the third week after infection), the serum concentrations of IFN-γ were shown to correlate with serum viral RNA loads and the severity of clinical signs (59). A role of NA and PRRSV-specific IFN-γ responses was highlighted for the protection after vaccination or primary infection (60, 61). In particular, a protective role of NA was demonstrated by passive transfer to adult and non-adult pigs (62, 63). Similar results were observed in the arterivirus infections of horses and mice (61). The findings are less clear and definitely contentious with respect to virus clearance after PRRSV infection. Thus, a recent experimental study (64) showed that viremia may coexist for weeks with NA and that, under some circumstances, heterologous neutralization may be more efficient than the homologous one. Also, a commercial inactivated vaccine was shown to evoke a vigorous post-challenge anamnestic NA response and no protection (65). On the whole, serum-NA and PRRSV-specific IFN-γ secreting cells (SC) do not fully depict the immune effector functions related to protective immunity (3), which probably underlies the accumulation of contradictory reports. In particular, some virus strains may fail to induce a satisfactory NA response to themselves, whereas other PRRSV strains can induce cross-reacting NA. This is probably related to the “glycan shielding” status, i.e. the glycosylation of crucial neutralizing epitopes (66). On the whole, *in vitro* neutralization should be further investigated, in that a NA response can be either associated with protection (62) or coexist with viremia for a long time (64). This probably means that neutralization measured *in vitro* may or may not correspond to an effective immune effector function *in vivo*. Also, classical anamnestic antibody responses do not occur in PRRSV-infected or vaccinated animals (3). Finally, there is strong evidence that immune activation measured *in vitro* (assay of PRRSV-specific, IFN-γ SC) is not dependent on the genetic divergence of the PRRSV strains used for immunization of pigs and recall *in vitro* tests, respectively (67).

**Figure 3:**
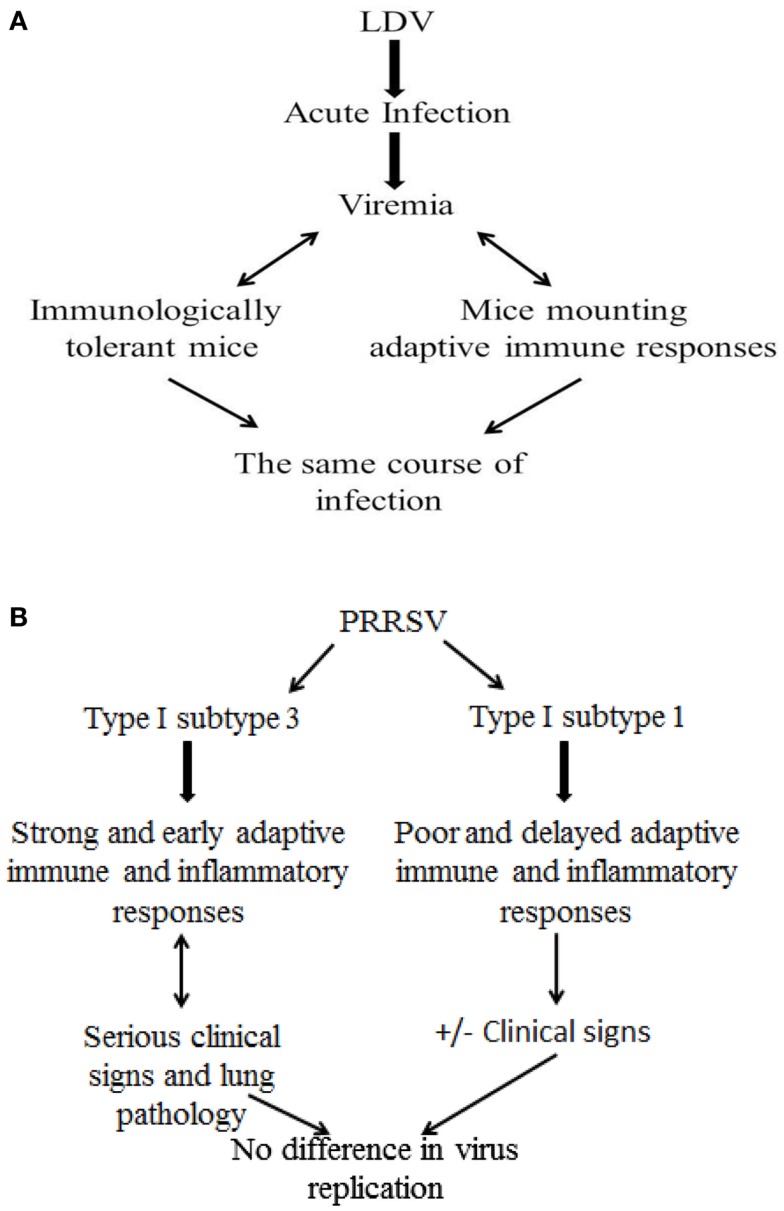
**Role of adaptive immune responses to established arterivirus infections: LDV and PRRSV models**. **(A)** LDV model in mice. **(B)** PRRSV model in pigs.

The above findings about PRRSV infection should be offset against studies of the closely related lactate dehydrogenase-elevating virus (LDV), a macrophage-tropic arterivirus of mice. After the acute phase of infection, LDV levels in the blood remain high for the rest of the host’s life (68). Viremia is maintained at a constant level, and the same course of the infection is observed in immunologically tolerant mice and in those mounting virus-specific humoral and cell-mediated responses (69). This implies that adaptive immunity plays little, if any, role in the control of established arterivirus infections in the murine model. This tenet would be confirmed in the PRRS model after infection with a virulent, type I, subtype 3 Belarus PRRSV strain (Figure 3). This gives rise to greater clinical signs and lung pathology with an enhanced and earlier adaptive immune response (IFN-γ SC and antibody), compared with subtype 1 strains (48). The effects can be accounted for by an enhanced inflammatory response, and not by higher virus replication (48). The same results were reproduced by immunization of pigs with DNA vaccines containing open reading frame (ORF) 5, 6, and 7 of PRRSV. An exacerbation of the disease after challenge was observed in DNA-immunized pigs that mounted a greater and earlier antibody response and rise of PRRSV-specific, IFN-γ SC, accompanied by higher levels of IL-1β in serum, compared with control, non-vaccinated animals (70). Apart from adaptive immunity, the lack of PRRSV-susceptible macrophage cells at some time during infection (34, 71, 72) could play an important role in the decay of viremia and in the further control of virus infection. On the whole, the above findings would imply that established PRRSV infections could be effectively controlled following (a) the lack of virus-permissive macrophages and (b) the development of macrophages refractory to productive infection as a result of an effective innate immune response. Also, an effective control of the inflammatory response could play a crucial role in preventing serious clinical signs in PRRSV-infected pigs.

### NK memory cells: The missing link?

The innate immune response to PRRSV could be displayed in different forms. Among these, the recent report about the antiviral activity of some peptides of the defensin and cathelicidin families in the PRRS model (42) deserves utmost attention; their antiviral activity could underlie, in fact, a potent mechanism of protection at the very sites of virus entry in the upper respiratory tract.

The innate, non-specific IFN-γ response (65), allegedly by CD3−CD8α+ NK cells (73, 74), could play an important role during PRRSV infection, in view of the constitutive expression of IFN-γ in swine PBMC (75). Interestingly, in the authors’ experience, the discrimination between virus-specific and non-specific IFN-γ responses in PRRSV-infected pigs gets ill defined whenever a cryolysate of non-infected cells is used as control antigen representing damage-associated-molecular patterns (DAMPs); the *in vitro* IFN-γ response to DAMPs is often equal or greater than the recall response to PRRSV (38).

Most important, recent data showed that an “education” of the innate immune system is possible. Thus, the system could better react to a second encounter with a virus by more effective effector functions (76), in contrast to established dogmas about the lack of memory in the innate immune response. In this respect, the strong circumstantial evidence about “adaptation” of pigs to field PRRSV strains could be set into a credible conceptual framework. Which mechanisms could be surmised? NK cells can acquire some form of immunological memory, and enhanced NK functions can be displayed during secondary, compared to primary exposure (76). This tenet is in line with the selective education process of NK cells and their clonal-like expansion during virus infections. Is this model relevant to PRRS in swine? Although only speculations can be made for the time being about the role of NK memory cells in PRRSV infection, there is indirect evidence in agreement with this latter hypothesis. Thus, PRRS-naïve, non-adult pigs experience a dramatic decrease of NK cell-mediated cytotoxicity very early after PRRSV infection on farm, which is related to massive virus replication and sustained viremia (77). In older pigs, the NK response could become more robust. In our experience, a substantial upregulation of CD3−CD8α+, allegedly NK cells (78) takes place early after infection with an attenuated PRRSV strain, which is coincident with the decay of viremia (40). CD3−CD8α+, allegedly NK cells, could also play a crucial role in local antiviral immunity in PRRSV-positive endometrium (28). Furthermore, after exposure to a field PRRSV strain, the same lymphocyte population was shown to be upregulated in animals inoculated with a live attenuated PRRS vaccine, as compared with the control, non-vaccinated ones (79). This finding was correlated with an increased expression of inflammatory cytokine genes, a prompt increase of growth hormone and greater early cortisol response with respect to controls. Vaccinated pigs also showed a more efficient shut-off of the TNF-α, IL-6, and IL-10 responses in the late phase of natural infection (80). These results imply that innate immune mechanisms maintain their importance for the host’s protection after primary infection or vaccination. Finally, a stepwise maturation of NK cell functions was shown during *in vitro* recall responses of PBMC from PRRSV-infected pigs (81).

### Roles of innate and adaptive immunity

There is a general consensus in the scientific community about an active suppression of innate immune responses early after infection by many PRRSV strains. However, there is also evidence in the long term of a reduced susceptibility to a further PRRSV infection after both homologous and heterologous challenge exposure (64). These findings may outline a new scenario, whereby failure or sub-optimal expression of innate immunity would often take place in the early phase of PRRSV infection, but not in the later ones, in agreement with the final virus clearance in the infected host (82). This concept can be conducive in our opinion to better disease control strategies. As a caveat, the above data obtained in studies of experimental infection may not be totally relevant to a field scenario, where subsequent “waves” of subclinical infection may take place over several weeks in non-adult pigs in PRRS unstable sow herds (38, 83). In this respect, the authors believe that the aforementioned exposure to microbial agents and airborne LPS, as well as to airborne barn dust (84) on farm is likely to play a crucial role. This could deeply modify fundamental parameters of the host/virus relationship and contribute to long-term persistence of PRRSV by (a) impairing and/or delaying the development of crucial immune responses, and (b) sustaining a poorly controlled inflammatory response.

Innate immunity should be complemented by PRRSV-specific, antibody, and cell-mediated responses, which were correlated with a better performance of PRRS vaccines against homologous, compared with heterologous challenge (85). Yet, we believe that even a homologous vaccine or field strain is not likely to induce a protective response if parameters of innate immunity are not adequately stimulated. This probably accounts for reports about equal or better virological protection by some heterologous PRRSV strains (64), and the existence of outright “immunotypes” of the virus (44). This implies that “immunotypes” of PRRSV better stimulating innate immunity are more likely to induce protection in pigs.

## The Efficacy of Vaccines

In terms of clinical protection and prevention of economic losses, farm hygiene, biosecurity measures, and welfare-friendly housing conditions in a PRRS-stable farm can be conducive to a favorable outcome of PRRSV infection. Pigs can “adapt” to field virus strains and show good productive figures in both reproductive and growing/finishing units, despite an ongoing PRRSV infection (11). In this scenario, PRRS vaccines should provide an added value by inducing effective immunity to field PRRSVs. Vaccines should prevent infection, virus-driven immunosuppression, growth check, virus transmission, and disease onset, as possible efficacy parameters. Also, PRRS vaccines are requested in the breeding sector to prevent transplacental transmission of the virus to pig fetuses. Most important, vaccines should provide an advantage in terms of disease control over the natural course of the infection, which eventually results in PRRSV clearance (82). In this respect, there is evidence that a commercial live virus vaccine may not substantially reduce virus transmission, since the relevant *R*_0_ values (basic reproduction number, the number of cases one case generates on average over the course of its infectious period) can exceed the critical 1.0 threshold (86). Regardless of this feature, vaccines should be conducive to a better disease status in PRRS unstable farms. In general, the performance and the role of both inactivated and live-attenuated PRRS vaccines have been controversial and not easily intelligible. A substantial efficacy of live attenuated, as opposed to inactivated PRRS vaccines, was advocated in some studies (65, 87). Yet, the efficacy of inactivated vaccines against reproductive disease was demonstrated in other studies in terms of piglets born alive and healthy, as well as of reduction of premature farrowings, abortions, and increased farrowing rates (88, 89).

Live-attenuated vaccines show variable levels of efficacy: both genetic relatedness to field strains and (more probably in our opinion) “immunotype” of vaccine and challenge strains might account for such outcomes. Regardless of this issue, the absence of a possible reversion to virulence (90) and of immunosuppressive properties should be much sought after features of live attenuated vaccines. On the basis of the findings reported about PRRSV immunotypes (44), a proper screening *in vitro* of candidate vaccine strains should include a thorough evaluation of the cytokine responses by leukocytes (PBMC, macrophages, dendritic cells), including in particular IL-10, type I, and type II IFN responses. Interestingly, a clear correlation was observed between induction of IL-10 *in vitro* and poor performance of an attenuated vaccine, regardless of the genetic similarity between vaccine and challenge strains, and development of NA before challenge (91).

*In vivo*, an attenuated vaccine strain should give rise to moderate IFN-γ responses in serum in the first week after parenteral injection, with little if any IL-10 response. Viremia should be over in a couple of weeks and cross-reacting NA should be detected within 3–4 weeks after injection. On the whole, the balance between induction of IL-10 and development of IFN-γ-SC should be taken into account for a proper evaluation of a candidate vaccine strain (91). Also, the presence of differentiating infected from vaccinated animals (DIVA) features would be an important added value for such vaccines, which has already prompted some research in this area. The demonstration of disposable segments in NSPs (92) lends support to the development of live-attenuated DIVA vaccines for PRRS. A modified live virus (MLV) containing a positive marker green fluorescent protein (GFP) was reported as well (93).

In general, the efficacy of vaccines may be negatively affected by the extreme variability of PRRSV strains, in terms of both genetic relatedness and virus “immunotype.” Interestingly, vaccine-induced protection cannot be restricted to genetic similarity between vaccine and challenge strains, and even against a closely related virulent strain, the protection can be only partial (94). Vice versa, immunization with a type I PRRSV vaccine can provide substantial protection against challenge with a highly virulent type II strain (95). Also, circumstantial evidence on farm does not point at a clear-cut correlation between nucleotide divergence of isolates from the vaccine strains and clinical impact of PRRS under field conditions. It should be stressed that clinical protection of PRRS-vaccinated pigs may substantially differ from virological protection (79), which means that virus loads in tissues do not account for severe clinical symptoms during PRRSV infection. On the basis of the above findings, one might wonder if the better performance of many vaccines against homologous PRRSV strains may be partly correlated with better adaptation of the host to PRRSV strain-specific regulation of innate immune responses.

Another crucial factor related to vaccine efficacy is the timing of vaccination with respect to the exposure to PRRSV field strains, having in mind that vaccination is often applied on farm to PRRSV-infected animals. In the authors’ experience, the efficacy of a live-attenuated vaccine under such conditions on farm may be negligible (96). Interestingly, a substantial efficacy of the same commercial vaccine was reported instead in another study, in which it was administered to PRRS-free young pigs, later moved to a PRRSV-infected site (79). The use of vaccines in subjects experiencing a wild-type infection deserves due consideration. Favorable effects of emergency vaccination were reported if a MLV vaccine was administered on the same day of contact exposure to PRRSV-infected animals (97). As for the vaccination of PRRSV-infected pigs, results are less clear. Under very favorable experimental conditions (wild-type virus homologous to the MLV vaccine), virus transmission and persistence were reduced in a late phase of infection (beyond 4 months) by one or more shots of vaccine (98). However, no significant difference was observed in terms of clinical signs and virus persistence after a heterologous infection; there was just a slight improvement in terms of virus transmission (99).

These and other results confirm a decreased susceptibility to PRRSV infection in convalescent animals. Vaccines could improve such a process in the long term, which may account for the favorable reports about disease-control schemes based on both vaccination and management of pig flow (100). Notice, however, that the presence of long-lasting viremias in infected, non-adult animals is conducive to further spread of infection whenever disposable needles are not used for vaccination of PRRSV-viremic subjects under field conditions.

On the basis of the above data, five major issues should represent as many priorities for a correct evaluation of any immunizing product against PRRS:

- Which parameters of protective immunity should be adopted?
- Can they be adequately stimulated by vaccines?
- If PRRSV is indeed immunosuppressive, can we develop MLV vaccines devoid of such side effects?
- Can inactivated vaccines be made more effective by proper adjuvants?
- Are PRRS vaccines effective, useless, or noxious vis-à-vis concomitant exposure to field PRRSV strains?

In the authors’ opinion, correct solutions to these problems will be conducive to a sound and meaningful evaluation of existing and new PRRS vaccines. In this respect, the findings generated by experimental studies should be complemented whenever possible by field data. Yet, as suggested in a previous study of our group (96), no single field trial of PRRS vaccine products is likely to provide conclusive efficacy data, pending a clear definition of the environmental parameters affecting the results, and relevant standardization features of field vaccine studies.

### New vaccines

It is far beyond the scope of this review to perform a detailed evaluation of the extensive research efforts in the area of PRRS vaccine research. Therefore, only some outstanding results will be taken into account and analyzed in the light of the above five major issues; for more information, the reader is referred to updated comprehensive reviews (3, 101).

In general, the efficacy of PRRS vaccines is affected by the immune evasion strategies of PRRSV; therefore, new vaccines will have to overcome these negative features and induce a broad immune response in vaccinated animals (66). This goal can be pursued by means of effective immunogens and/or new ways of antigen delivery, formulation, and injection. In particular, it is questionable whether vaccines can successfully antagonize the PRRSV-induced negative regulation of the immune response at the time of infection (102).

Regardless of the vaccine type to be developed (live vs. inactivated), recent findings suggest that two areas of research deserve particular attention: mucosal vaccines and bromo-ethylene-imine (BEI)-inactivated vaccines. As for mucosal vaccines, the results based on type II MLV strain VR2332+ whole cell lysate (WCL) of *Mycobacterium tuberculosis* (*mtb*) administered intranasally (103) are remarkable in terms of clinical protection (symptoms, lung lesions, and weight gain) and reduced viremia after a heterologous challenge, which highlights the importance of “immunotypes” and antigen presentation as crucial parameters for vaccine efficacy, rather than genetic relatedness between vaccine and challenge strains. Vaccine efficacy was correlated with a global increase of T helper (Th)1 responses (IFN-γ, IL-12, IFN-γ SC), NK cell frequency, and function, NA response, as well as with reduced production of IL-10 and Treg cells (103, 104). Also, there was an increased proliferation of CD8+ lymphocytes on restimulation among lung and peripheral blood mononuclear cells, and an increased content of nitric oxide in the lung homogenate (105). Similar favorable results were observed following intranasal inoculation of poly(lactic-*co*-glycolic acid, PLGA) nanoparticle-entrapped killed PRRSV (106). On the whole, the above results suggest that mucosal administration of PRRS vaccines with proper adjuvants can give rise to effective immune responses.

The development of inactivated vaccines can be based on proper inactivation procedures of PRRSV with ultraviolet light, BEI, and gamma irradiation, i.e., methods with a main effect on viral genome, preserving PRRSV entry-associated domains (107). The preservation of such critical domains is correlated with good immunizing properties of BEI-inactivated, oil-in-water PRRS vaccines, characterized by a good NA response and a significant reduction of viremia after challenge (108); partial protection against a heterologous strain can be demonstrated as well (72). Therefore, it is conceivable to boost the immunity induced by attenuated vaccines with properly inactivated vaccines of high antigenic mass (72). The immunizing properties of killed vaccines could be also enhanced by TLR ligands like polyriboinosinic polyribocytidylic acid (poly I:C) and CL097 (a derivative of the imidazoquinoline compound R848), with lighter clinical signs and lower viremia after infection as compared to control vaccine and challenge control groups (109).

The development of PRRS recombinant vaccines has been seriously hampered by uncertainty about the viral targets of protective immunity (3) and by the serious problems in obtaining a gene-deleted attenuated strain (66). A more promising, feasible approach is the preparation of chimeric MLV vaccines, based on the exchange of one or more ORF regions between a virulent and an attenuated PRRSV strain (110). Whereas DNA vaccines usually require multiple injections and are thus not compatible with mass vaccination programs, recombinant proteins expressed in safe, suitable vectors could be a possible option in the future to induce broad NA and/or cell-mediated responses to promiscuous T-cell epitopes (5). These products could be used for either primary or recall vaccination programs. In this respect, some viral vectors (pseudorabies virus, poxvirus, adenovirus, transmissible gastroenteritis virus, TGEV) have the capability of expressing high levels of heterologous PRRSV genes; these vectors can induce PRRSV-specific antibody and partial protection against a challenge infection (111).

Several adjuvants for PRRS vaccines were investigated in previous studies. As stressed in a previous review (112), both interleukin-2 and CpG oligodeoxynucleotides (CpG ODN) were shown to enhance the protective efficacy of PRRS vaccines in challenge models. Interestingly, these results refer to DNA and killed vaccines, whereas no adjuvant was shown to enhance the protective efficacy of PRRS MLV vaccines for parenteral injection (112). Vaccines targeting dendritic cells or suppressing negative regulatory functions of Treg cells could be of major importance, but probably cost-prohibitive for immunization of swine (110).

## Immunomodulation: Conceptual Basis in the PRRS Model

The uncertainty about the viral targets of protective immunity and the poor definition of clear immunological correlates of protection are as many serious hurdles in the development of novel effective PRRS vaccines (3). Thus, there is a case for complementary disease control strategies based on the modulation of innate immune effector functions, which are conducive to both clinical and virological protection. The issue of an immunomodulation strategy for PRRS is very complex, since PRRSV itself is definitely an immunomodulator (suppression of IFN responses, stimulation of polyclonal IgG responses, etc.). In the authors’ opinion, three aims should be considered within an immunomodulation strategy:

1. To maintain the homeostatic regulatory functions of IFNs during PRRSV infection.
2. To antagonize the PRRSV-driven amplification of the inflammatory response to LPS.
3. To boost antiviral innate immunity.

In other words, combating PRRSV-induced negative regulatory effects in the early phases of infection (102) defines the scope of an immunomodulation strategy for PRRS. This could properly stimulate CD3−CD8α+, allegedly NK cells in endometrium by target cell receptor-specific immunoconjugates (28).

Aims 1 and 2 should be meant in terms of clinical rather virological protection. Aim 3 could be relevant to both. Non-adult pigs, as well as pregnant sows would be obviously the main targets of an immunomodulation strategy. An immunomodulator for PRRSV infection could have been inadvertently under use for some years, because of a possible role as immunomodulators of inactivated porcine circovirus type 2 (PCV2) vaccines. The evidence underlying this statement is both diverse and complementary. First, circumstantial evidence in the field shows that the dramatic decline of PCV2-associated pathologies after the introduction of inactivated PCV2 vaccines has often gone along with a decrease of clinically overt respiratory PRRS and PRRSV-related disease cases. Second, the findings of a field trial showed an improved clinical response to PRRSV infection in PCV2-vaccinated pigs (96). In the same study, the significant downregulation *in vitro* of TNF-α gene expression vis-à-vis PRRSV in leukocytes of PCV2-vaccinated pigs pointed at a regulatory function of PCV2 vaccines on the immune response to PRRSV (96). In another field study (113), a PCV2 vaccine based on recombinant ORF2 was shown to improve the clinical score, the daily weight gain, and the time to market in a PRRSV-infected farm, affected by PRDC. In a PCV2 vaccine trial based on recombinant ORF2 (114), more lung samples from placebo-treated animals than from vaccinated animals were found to be positive for *Mycoplasma hyorhinis* and PRRSV. Obviously, the authors do not rule out an indirect effect of PCV2 vaccines; an effective immune response to PCV2 could curtail the substantial synergism between PCV2 and other pathogens in the framework of PRDC, as confirmed by the overlapping post mortem findings of PCV2 infection and PRDC (115). Further studies are needed to elucidate and confirm these findings, as well as to clarify the role of PCV2 vaccines in disease control programs in swine herds.

A typical Th1 cytokine like IL-12 can significantly decrease PRRSV titers in lungs and blood of infected animals and prevent significant growth retardation; also, IL-12 induces IFN-γ in PAM and reduces PRRSV titers *in vitro* (116).

### The interferon system as target and tool of an immunomodulation strategy

The IFN system could be an important target of an immunomodulation strategy for PRRS, having in mind the negative regulatory functions of PRRSV on this crucial arm of the innate immune system (41). PRRSV is susceptible to the direct antiviral mechanisms displayed by IFN-α (117) and IFN-γ (118). Most important, IFNs were shown to reverse distinct immunosuppressive functions of PRRSV. Thus, IFN-α can block *in vitro* the development of Treg cells induced by co-culture of lymphocytes with PRRSV-infected dendritic cells (43). *In vivo*, positive effects were observed in pigs injected with a non-replicating human adenovirus 5 vector expressing porcine IFN-α, in terms of lower febrile response, lesser involvement of lungs, delayed viremia, and antibody response after challenge with PRRSV 1 day later (58). Yet, the peak and duration of viremia were not significantly different between treated and control animals (58), which points once again at the profound discrepancies between clinical and virological protection.

A substantial stimulation of antiviral innate immune responses can be pursued by low-dose, oral IFN-α treatments (119). Human IFN-α can be conveniently administered to pigs since the protein is biologically active on porcine cells and compatible with freeze-drying (120). The biological effects are exerted following an interaction with the oral lymphoid tissues (121). Therefore, IFN-α does not need to resist proteases and to be absorbed through the gut after passing the stomach in an active form (acid-stable cytokine).

Owing to the above, two field trials of oral, low-dose human IFN-α treatment (10 IU/kg b.w./daily) were carried out by our group in problem herds, affected by recurrent outbreaks of PRRS; the results of these trials were reported in a previous review paper on IFN-α (122). The first trial was carried out in a large multi-site, farrow-to-weaning herd, affected by a typical, clinically serious respiratory form of PRRS in pigs of about 40 days of age, with the demonstrated involvement of both PRRSV and PCV2. The oral, low-dose IFN-α treatment caused a significant reduction of dead piglets and “poor-doers” (*P* < 0.01); the treatment also caused a much greater average daily weight gain from 22 to 86 days of age (Table 1). Similar results were obtained in trial 2, carried out in a farrow-to-finish herd also affected by respiratory PRRS (Table 1). Following these trials, we were requested by swine practitioners to perform this treatment in two farms heavily affected by reproductive PRRS. In the former, the treatment was carried out on sows housed in farrowing crates, whereas in the latter, the treatment was applied to all pregnant sows regardless of the pregnancy phase. In both cases, there was clear circumstantial evidence of a great reduction of both abortions in sows and mortality in suckling piglets within 2 weeks after administration of IFN-α, even though the lack of control groups did not enable us to evaluate the real efficacy of the applied treatment. In particular, we cannot rule out that IFN-α somehow stimulated a rapid development of PRRSV-specific immunity, providing protection to later farrowing animals before they reached the critical late-gestation period of maximum sensitivity to PRRS disease.

**Table 1:** **Oral, low-dose IFN-alpha treatments on farm for respiratory PRRS**.

(A) Trial 1					

Groups	Piglets	Mean weight at 22 days (kg)	Mean weight at 86 days (kg)	ADWG (kg)	Dead piglets and “poor-doers” (%)
IFN α-treated	280	5.6	37.1	0.48	0.3*
Control	280	5.6	33.8	0.44	4.5*

**(B) Trial 2**					

**Groups**	**Piglets**	**Mean weight at 24 days (kg)**	**Mean weight at 72 days (kg)**	**ADWG (kg)**	**Dead piglets and “poor-doers” (%)**

IFN α-treated	458	7.20	28.61	0.446	0.7*
Control	430	6.98	27.38	0.425	3.5*

*P < 0.01 (Fisher’s exact test).

*The two trials were carried out in as many herds affected by recurrent respiratory PRRS and PCV2-associated pathologies after weaning. Freeze-dried human lymphoblastoid IFN-alpha was mixed with the starter feed of piglets at weaning to provide 10 IU/kg of body weight for 15 days in a row, on the basis of the mean weight at weaning and the expected growth rate of piglets. The control groups received the same starter feed without IFN-alpha. The whole report in Italian language is available at: http://www.izsler.it/izs_bs/ftp//doc/Archivio/2002/2002Agosto.pdf*.

*ADWG, average daily weight gain*.

On the basis of these field findings, we decided to investigate their possible reproduction under the “clean” conditions of an experimental infection of weaners with an EU PRRSV strain inside isolation facilities, with little if any infectious pressure exerted on the pigs. In this case, we observed no overt disease symptoms, and only minor clinical differences were shown between IFN α-treated and control piglets; there was just a significant decrease of pyrectic days (*P* < 0.05) and a transient decrease of circulating CD8+ T and NK cells for a possible homing to PRRSV-infected tissues, not observed in control pigs (data not shown). Interestingly, the significantly fewer days of fever in IFN α-treated pigs was not related to the duration of viremia, in agreement with the aforementioned results of adenovirus type 5 vector expressing porcine IFN-α (58). In a completely different context, the same discrepancy between viremia levels and improvement of clinical score after an oral, low-dose IFN α-treatment was observed in feline immunodeficiency virus-infected cats (123). On the whole, the comparative evaluation of trials on farm and in isolation facilities indicated that the control action of IFN-α was not exerted on the viral infection, but rather on the mutual interactions between PRRSV, environmental bacteria, and/or airborne LPS (17), obviously lacking in isolation facilities. The absence of a direct antiviral effect of low-dose IFN-α is also in line with the possible enhancement of PRRSV infection in IFN α-treated PAM via upregulation of the sialoadhesin receptor (124).

### Effector mechanisms of oral, low-dose IFN-alpha treatments

The authors believe that the LPS/PRRSV synergism (17) is pivotal to understanding the activity of low-dose, oral IFN-α. As shown by our *in vitro* data (120), very low concentrations of hIFN-alpha (0.5–5 IU/ml) downregulate CD14 expression in swine PBMC, as opposed to higher concentrations. This may dramatically affect signaling by LPS/LPS binding protein, and the released CD14 may be a potent scavenger of LPS. Most important, our laboratory has recently demonstrated a potent role of IFN-α in the control of inflammatory cytokine responses through the release of secondary mediators from IFN α-treated tonsil cells, which underlies the rationale for an oral, low-dose treatment with type I IFNs (125). The anti-inflammatory control actions of IFN-α are also directly exerted *in vitro* on epithelial cells at both moderate and low concentrations (125).

In this scenario, IFN-α could prevent a negative clinical outcome following exposure to PRRSV and bacterial LPS by checking abnormal inflammatory cytokine responses. This is in line with the observed correlation between the serious clinical course of PRRSV infection and a strong inflammatory response (48).

The control action of IFN-α on inflammatory cytokines could be performed through different, non-mutually exclusive, dose-dependent pathways: mRNA stability control by tristetraprolin (TTP) induction (126), Tyro3, Axl, and Mer (TAM) receptor-mediated activation of suppressor of cytokine signaling (SOCS) proteins (127) through type I IFN receptor (IFNAR-1) signaling (128), downregulation of CD14 expression (120).

## Concluding Remarks

On the whole, the accumulated findings indicate that PRRS should be considered as a peculiar model of host/virus relationship, where the final clinical outcome is actually determined by the combination of virus infection, bacterial co-infections, pig and virus genetic features, and fundamental environmental data (hygiene and welfare conditions). In this scenario, PRRS has evolved in its history from a subclinical infection in non-lean pigs to a sometimes serious disease in lean type pigs. The infectious pressure on farm and the intensity of exposure to airborne LPS are often of major importance for an unfavorable clinical outcome of the infection.

The pig eventually gets rid of an established PRRSV infection. This is mainly due in the authors’ opinion to as yet undefined features of innate immunity, which are also likely to play an important role in protection against primary infection and/or re-infection. This kind of protection can be also afforded by adaptive immune responses, in particular by NA. Vaccines may promote the development of effective innate and adaptive responses. This is more likely to happen if vaccination is carried out in PRRSV-free animals, whereas advantages are dubious in PRRSV-infected animals. If vaccination of PRRSV-free animals should not be possible, priority should be given to good farming practices (all in–all out and all-forward practices, hygiene, strict control of animal concentrations, limitation of nursing practices) to reach or maintain a “PRRS-stable” status; this can be associated with satisfactory clinical conditions and productive performance. The foundation of this working scheme is possibly the stepwise maturation of the innate immune response as a result of a controlled exposure of animals to field PRRSV strains.

To achieve a “PRRS-stable” status, immunomodulation could be conveniently associated with proper farm management measures. This is true in particular for pig farms where (a) fundamental logistics and infrastructure features do not allow for a rapid enforcement of sound hygienic measures and (b) recent anamnestic data confirm heavy losses due to circulating PRRSV strains.

Positive clinical results can be obtained on farm by oral, low-dose IFN-α treatments, as opposed to the negligible outcome under experimental conditions in isolation facilities. This indirectly confirms the role of environmental bacterial agents in clinically overt PRRS. Accordingly, the regulation of the inflammatory response by oral IFN-α is likely to play a crucial role in preventing a clinically serious outcome of PRRSV infection.

On the whole, a sound combination of clinical surveillance, good farming practices, welfare-friendly conditions, and immuno-prophylaxis is badly needed for an effective control of PRRS in the long term. In this scenario, immunomodulators could be of some use in “problem” farms showing recurrent PRRS outbreaks. At the same time, the fundamental mechanisms of PRRSV clearance and protection against re-infection still pose a challenge to the scientific community. This will probably demand the development of new working hypotheses and experimental models beyond established dogmas and outdated investigation schemes.

## Conflict of Interest Statement

The authors declare that the research was conducted in the absence of any commercial or financial relationships that could be construed as a potential conflict of interest.
